# Placebo-controlled trial of oral amantadine and zolpidem efficacy on the outcome of patients with acute severe traumatic brain injury and diffuse axonal injury

**DOI:** 10.22088/cjim.13.1.113

**Published:** 2022

**Authors:** Sajad Shafiee, Saeed Ehteshami, Mahmood Moosazadeh, Saeed Aghapour, Kaveh Haddadi

**Affiliations:** 1 Department of Neurosurgery, Orthopedic Research Center, Mazandaran University of Medical Sciences, sari, Iran; 2 Gastrointestinal Cancer Research Center, Non-communicable Diseases Institute, Mazandaran University of Medical Sciences, Sari, Iran; 3 Department of Neurosurgery, Faculty of Medicine, Mazandaran University of Medical Sciences, Sari, Iran

**Keywords:** Amantadine, Zolpidem, Traumatic brain injury, Diffuse axonal injury

## Abstract

**Background:**

A constituent of diffuse axonal injury (DAI) is supposed to be present in about 1/3 of all severe traumatic brain injury (TBI) as specified by pathologic documents. Diffuse axonal injury is categorized by extensive injury to axons in the brain. A rise in the incidences of TBI, and the limited study to verified effect of drugs like amantadine and zolpidem in improving the consciousness levels of patients with acute traumatic brain injury with axonal injury enthused us to initiate this study in the acute TBI patients.

**Methods:**

In our randomized, controlled trial involving patients with acute severe TBI, we studied 66 patients in 3 groups. Group 1 (n=22) received oral amantadine, Group 2 (n=22) received oral zolpidem, whereas group 3 (n=22) received placebo, the first 8 days after injury respectively. The primary outcome measures included GCS (Glashow coma scale) through the initial admission, a complete medical history was recorded, and each patient had a meticulous physical and neurological investigation.

**Results:**

We found that the administration of amantadine in an acute phase after injury improved the rate of patients GCS and GOS (Glasgow Outcome Scale) compared with zolpidem and placebo groups, but without any significant statistical difference.

**Conclusion:**

Our results has emphasized that because amantadine has intense biochemical effects on several ways, it appears to be beneficial in acute period after DAI-associated TBI.

Traumatic brain injury (TBI) sometimes is a disastrous experience that has distressing familial and societal penalties. TBI is the commonest source of death and debility in young age (1, 2, and 3). The severe injuries can cause chronic disorders of consciousness. Today, the World Health Organization emphasizes that TBI will progress as the main reason of death and disability. Up to fifteen percent of severe traumatic brain injury patients are released with vegetative state, a situation in which there is restlessness and degree of conscious awareness (3, 4, and 5). The initial Glashow coma scale (GCS) can fairly describe the primary neurological complaint; but, it cannot exactly define the prognosis. (6, 7). Surgical or medical management for TBI are centered on the nature of the damage. The main cause of coma induced subsequent head trauma is not clear. Maybe, subcortical white matter stimulates disconnection of brain functional pathways. Thus, the metabolic action among the cerebral cortex and the deeper areas (particularly thalamus area) of the brain might be detached. This interruption was characterized as diffuse axonal injury (DAI). 

The DAI was separated into three grades: 1. injury to parasagittal white matter 2. grade 1 plus local damage to the corpus callosum and 3. injury to the crus cerebri and brain stem in addition to grades 1 and 2 (6, 8). The main mechanism of TBI in these circumstances are of high-speed, long-duration deceleration (9-11). DAI is described by extensive damage to axons in the brain cortex, brain-stem and the cerebellum (9-11). DAI is related by a decrease in dopamine turn over because of the injury to the cellular structure of midbrain (12, 13). It is a historical issue that the anatomical site and mechanism of brain injury can lead to changes in the neurotransmitter’s metabolism. Up to now, several and various clinical studies about TBI were unsuccessful to classify the patients based on severity, mechanism and anatomical site of damage (11-17).

Dopamine, is an essential brain neurotransmitter. In the acute phase after TBI, the levels of catecholamine increase in the cerebrospinal fluid (CSF). Plasma norepinephrine has been revealed to associate with variations in the Glasgow Coma Scale (GCS) score and so will be possibly associated by the outcome of TBI (17-20). Drug’s effect on dopaminergic pathway has been described to be effective in refining cognitive function and consciousness state in patients with moderate to severe TBI. Amantadine can have effect on these chemical pathways and may preserve brain cell functions (19-22).

Amantadine is a water-soluble amine salt that is involved in synthesis and distribution of catechol amines in the CNS (central nervous system). It is quickly absorbed; however, it is not metabolized. Amantadine is defecated in the urine, and the removal half-life is up to 14.5 hours (23, 24). Amantadine induces the release of dopamine from neuronal cells, assists dopamine release by nerve stimulation, and suspends the re-uptake of dopamine by means of neurons (14, 24, and 25). It may also enhance the antagonist effects on NMDA (N-methyl-D-aspartate) receptor that might be related to its early neuro-protective properties after brain injury. Amantadine is typically suggested as an anti-Parkinson’s medication, and as an anti-viral mediator. In detail, amantadine was primary established as an anti-viral mediator and has confirmed effectiveness in the influenza disease prophylaxis (25).

Several animal trainings have similarly exposed the influence of amantadine on cumulative dopamine levels, particularly in the cortex of frontal lobe. Amantadine can pass the BBB (blood-brain barrier) simply and is quantifiable in the cerebrospinal fluid (CSF). Restricted studies have verified the valuable effect of amantadine on refining awareness state in acute phase of TBI (26, 27, and 28).

Zolpidem is a short-acting imidazopyridine class drug typically used for insomnia (29). It has been revealed to prompt unpredictable reactions in some patients with cognizance disorders, and improve cognitive abilities. Numerous studies presented that they can persuade high exciting retrievals in harshly TBI patients with consciousness disorders from different etiologies (29-34). A change in the activity of brain especially in prefrontal cortices, and some parts of basal ganglia, after zolpidem consumption has been informed in existing studies using SPECT (single photon emission computed tomography) (31), positron emission tomography (PET), and electroencephalography (EEG) (32, 35). Based on this ideal, some authors imagined that a diminished brain metabolism in the thalamus, striatum and prefrontal areas would be detected, which would recover subsequent zolpidem intake compared to placebo (35).

It is assumed that after brain damage, the brain tries to turn off its metabolism to protect neurons to reduce metabolic demands. Animal studies show that the areas of the brain with poor perfusion after brain damage have found better perfusion and increased GABA levels following the use of zolpidem. (28, 36, 37). A rise in the incidences of TBI and the limited study to validate the effect of drugs like amantadine and zolpidem in improving the consciousness levels of patients with acute traumatic brain injury with axonal injury enthused us to design this study in patients with acute TBI admitted in intensive care unit (ICU).

## Methods

**Protocol review: **The study was approved by the local institutional ethics committee (Code: IR.MAZUMS.IMAMHOSPITAL.REC.1398.119). The project was also recorded as a clinical study with the Iranian Registry of Clinical Trials (IRCT20191026045243N1). A written informed consent was obtained from all the study participants. All the investigational processes relating to human samples were directed with the firm observance of the guidelines of the Declaration of Helsinki. 

**Figure 1:**
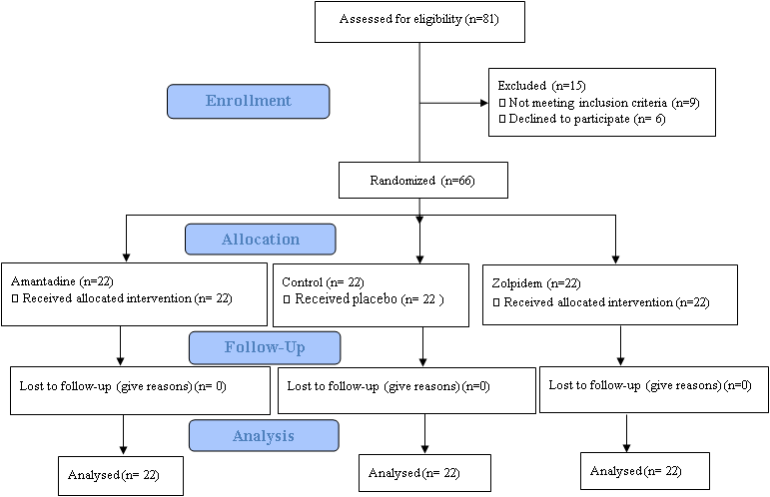
CONSORT 2010 Flow diagram

**Subjects: **The study population involved subjects enrolled over a 1.5-year period, who were straightly admitted via the Emergency Department with a GCS score of ≤ 8 and a TBI from a motor vehicle crash (MVC). The study was a single-blind, randomized, placebo-controlled study directed predominantly to obtain efficacy and safety data on the use of amantadine and zolpidem in acute TBI. After primary processing, 66 patients were selected among the total 81 patients. Random allocation software was used in blocking process and divided the patients in 3 randomizes groups. Group 1 (n=22) received oral amantadine, group 2 (n=22) received oral zolpidem, whereas group 3 (n=22) received placebo the first 8 days after injury, respectively. This management continued pending the anticipated outcome, death, or complication (Figure 1).

**Inclusion criteria: **All patients must join the study with diagnosis of diffuse axonal injury based on neurosurgery clinical and imaging characteristics and meet the subsequent criteria: 1. 15–75 years of age. 2. GCS score of ≤ 8 in the first day of trauma. 3. Patients without any focal significant hematoma in brain CT scan obligate surgery. 4. Patients not to have had an identified life-threatening illness before head injury. 5. A legal illustrative or protector of the subject gave an on paper informed consent on his or her behalf. 6. The subject was capable to get drug orally or by nasogastric tube. 

**Exclusion criteria**

1. Subjects, guardians or legal managers without tendency to collaborate with the study.

2. Subjects had received any other trial drug within one month before of injury. 

3. Subjects recognized to have had spinal cord injury with current deficits, congestive heart failure, severe ischemic heart disease, cancer, or any other severe diseases, in the opinion of the researchers that would disturb the valuation of treatment. 

4. Multiple traumas that in the estimation of the investigator would risk the valuation of therapy. 

5. Subjects with penetrating head injury.

6. Subjects with chronic steroid consumption.

7. Prior significant cerebral vascular event, TBI, brain tumor.

8. Patients with renal failure.

9. Pregnancy

10. Past history of allergy to amantadine and zolpidem

**Acute Neurological Management: **Through the initial hospital admission, all patients were treated agreeing to our neuro-trauma standard protocol, which includes similar anticonvulsant drugs and etc. Investigators filled the form comprising demographic features, the mean duration of mechanical ventilation, length of ICU stay, GCS at the admission, discharge or in-hospital fatality, Glasgow Outcome Scale (GOS), and the frequency of mortality in patients. In addition, examiners achieved the consent from the primary caregivers before beginning drug therapy. 

**Pharmacological agents: **Group 1 received oral amantadine, 200 mg/day, group 2 received oral zolpidem, 10 mg/day, whereas group 3 (n=22) received placebo the first 8 days after injury, respectively. Placebo was made by the drug manufacturer of starch in the form of special tablets to match appearance and shape and was ordered to patients.

**Outcome variables: **The primary outcome measures included GCS during the initial hospitalization, a full medical history was taken, and each subject had a detailed physical and neurological examination. Laboratory examinations, containing serum routine electrolyte, glucose, urea nitrogen, and creatinine were performed, likewise complete blood count and urinalysis. An electrocardiogram was also performed.

**Statistical methods: **Data entry and analysis was achieved with SPSS software Version 24. Quantitative variables were accessible as mean±SD and qualitative variables as numbers (percentage), frequency, mean, minimum and maximum.). Evaluation of demographic and clinical variables among the three groups was done by chi-square and Fisher's exact tests. Comparison of mean age and GCS score between the three groups done separately for each evaluate days was assessed by Kruskal-Wallis test. Also, the comparison of changes in the mean GCS score at different measurement times between the three groups (intergroup comparison) was evaluated by repeated measures analysis of variance. Comparison of changes in the mean GCS score at different measurement times in each group (intragroup comparison) was performed by Friedman test. Also, the effect size and percentage of changes between GCS score, admission time and discharge time were presented separately for each of the three groups. The p<.05 was reflected statistically significant.

## Results

Of the 22 patients in group amantadine, 15 (68.1%) of them were men and 7 (31.9%) were women. Of the 22 patients included in group zolpidem, 15 (68.1%) were men and 7 (31.9%) were women and 22 patients in group placebo, 15 (68.1%) of them were men and 7 (31.9%) were women. Based on the Fisher’s exact test, there was no significant difference between the sex of the patients in 3 groups (P=1.000). The mean age of patients in the group 1 was 40.72±14.58 years and the mean age in group 2 was 40.22±14.65 years and in group placebo was 47.40±16.01. According to ANOVA test, there was no significant difference between the age of the groups (P=0.220) (table 1).

**Table 1:** Demographic and clinical comparison in 3 group of study

**Variables**		**Intervention Group; n (%)**		**Placebo Group; n (%)**	**P-value**
		**Zolpidem**	**Amantadine**		
Gender	Male	15(68.2)	15(68.2)	15(68.2)	1.000
	Female	7(31.8)	7(31.8)	7(31.8)	
Diabetes mellitus	Yes	3(13.6)	2(9.1)	5(22.7)	0.578
	No	19(86.4)	20(90.9)	17(77.3)	
Hypertension	Yes	6(27.3)	4(18.2)	6(27.3)	0.569
	No	16(72.7)	18(81.8)	16(72.7)	
Kidney disease	Yes	1(4.5)	1(4.5)	0(0)	1.000
	No	21(95.5)	21(95.5)	22(100)	
Hyperlipidemia	Yes	3(13.6)	2(9.1)	1(4.5)	0.864
	No	19(86.4)	20(90.9)	21(95.5)	
Thyroid disease	Yes	0(0)	0(0)	0(0)	-
	No	22(100)	22(100)	22(100)	
Smoking	Yes	6(27.3)	3(13.6)	4(18.2)	0.637
	No	16(72.7)	19(86.4)	18(81.8)	
Age (mean±SD)		40.22±14.65	40.72±14.58	47.40±16.01	0.220

In group 1, the mean GCS at the time of admission was 5.40±1.14, and in group 2, the mean was 5.50±1.30 while in group 3 was 5.77±1.47. Based on the results of the analysis, there was no significant difference between the 3 groups in GCS at the time of admittance. The mean GCS at the time of discharge was 13.02±1.98, 12.26±1.25 and 12.98±1.86 in groups 1, 2, and 3, respectively. There was no significant difference between the GCS in 3 groups at the time of discharge.

**Figure 2:**
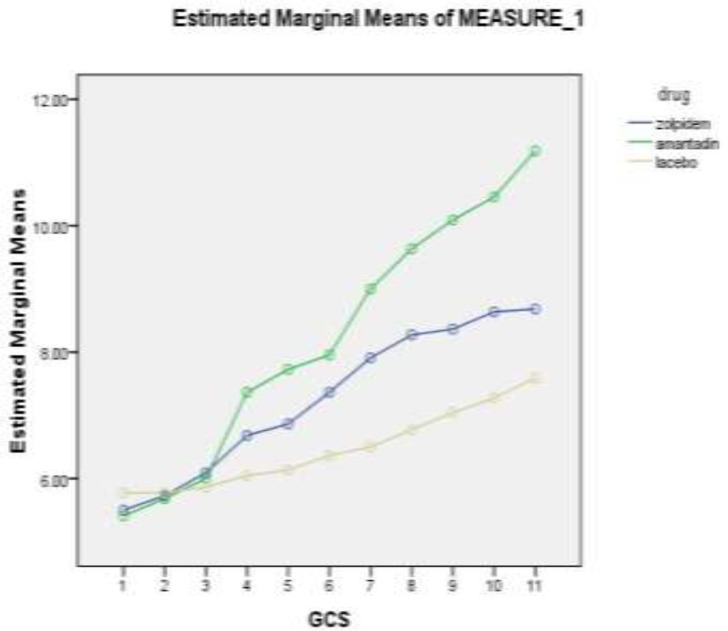
GCS change chart during study among zolpidem, amantadine and placebo patients

The highest mean GCS in the amantadine receiving group on the third day after discontinuation of the drug is 11.18±2.12. The highest GCS score on the third day after discontinuation was 15 and the lowest score was 7. In the same results as the zolpidem and placebo groups, the highest GCS average on the third day after discontinuation of the drug was 8.86±2.34 and 7.95±2.37, respectively. The highest and lowest GCS score on the third day after discontinuation of the drug in the zolpidem group is 13 and 3, respectively, and in the placebo group were 12 and 3 (figure 2).

Based on figure 2, the comparison of the mean GCS of individuals in 3 groups of zolpidem, amantadine and placebo was examined in the first 8 days of hospitalization and 3 days after discontinuation of the drug. Patients' scores have been increasing since the fourth day, with the largest increase in the amantadine receptor group. As can be seen, the GCS changes in all three groups are ascending. The same GCS is seen in all three groups in the first 3 days, but the trend has been increasing since the fourth day, especially in the group of patients receiving zolpidem and amantadine. This difference is evident in the group that received amantadine in this chart. Table 2 shows the comparison of the average GCS (±SD) of individuals in 3 groups of zolpidem, amantadine and placebo in the first 8 days of hospitalization and 3 days after drugs discontinuation (time 9-time 11).

**Table 2:** Comparison of the average GCS (±SD) of individuals in 3 groups of zolpidem, amantadine and placebo in the first 8 days of hospitalization and 3 days after discontinuation of the drug

**Variables**	**Intervention Group**		**Placebo Group**	**P-value -Kruskal-Wallis (between group)**	**P-value-repeated measure (between group)**
	**Zolpidem**	**Amantadine**			
Day 1 (Admission)	5.50±1.30	5.40±1.14	5.77±1.14	0.642	0.004
Day 2	5.72±1.45	5.68±1.32	5.77±1.57	0.972	
Day 3	6.09±1.47	6.00±1.60	5.86±1.45	0.771	
Day 4	6.68±1.46	7.36±1.76	6.04±1.55	0.046	
Day 5	6.86±1.39	7.72±1.85	6.13±1.64	0.011	
Day 6	7.36±1.64	7.95±1.96	6.36±1.73	0.023	
Day 7	7.90±1.97	9.00±2.09	6.50±2.17	0.001	
Day 8	8.27±2.09	9.63±1.86	6.77±2.30	<0.001	
Day 9	8.36±2.21	10.09±1.90	7.04±2.35	<0.001	
Day 10	8.63±2.42	10.45±2.08	7.27±2.54	<0.001	
Day 11	8.68±2.43	11.18±2.12	7.59±2.73	<0.001	
Effect size (between admission and discharge times)	1.63	3.39	0.87		
Change percent GCS score (between admission and discharge times)	57.8	107.03	31.5		
P-value-Friedman Test (within group)	<0.001	<0.001	<0.001		

Change percent GCS score (between admission and the days 11) were 57.8, 107.03, and 31.5 in zolpidem, amantadine and placebo group, respectively. Also as an important result; Effect size (between admission and day 11) was 1.63, 3.39, and 0.87 in those three groups, respectively. Showed the greater effect of amantadine in increase GCS in admitted patients. 

Finally, 1(4.54 %) patient in groups 1, 2 (9.09%) of group 2 and 2(4.54%) patients of group 3 died. But there was not a lost to follow- up in our study. Results of the Fisher’s exact test showed that there was no significant difference in the outcome of patients with TBI in 3 groups. The mean GOS in group 1was 2.01±1.12, 2.00±1.01 in group 2 and 2.02±1.11 in group 3. Finally, there was no significant difference between GOS in 3 groups at the time of admission (table 1). The duration of mechanical ventilation in group 1 was 19.14±14.27days, 20.21±15.49 days in group 2 and 22.12±15.88 in group 3. The t-test analysis showed no significant difference in the duration of mechanical ventilation between the 3 groups. The mean length of hospitalization was 27.12±16.88, 28.31±17.77 and 30.62±17.47days in three groups, respectively without any significant difference between them (table 3).

Both drugs were well tolerated at a dosage used throughout the study selected based on previous studies. There were no thoughtful adverse side effects, and both drugs look to be relatively safe. No patient was required an adjustment in drugs dose during the study because of an even slight unpleasant side effect. There were no major alterations in lab values through the study.

**Table 3:** Relationship of mean and standard deviation of GOS, duration of mechanical ventilation and hospitalization in the 3 groups

**P Value**	**Control Group**	**Treatment Group**		**Variable**
		**Zolpidem**	**Amantadine**	
0.52	22.12 ± 15.88	20.21 ± 15.49	19.14 ± 14.27	Duration of Mechanical ventilation
0.49	30.62 ± 17.47	28.31 ± 17.77	27.12 ± 16.88	Length of hospitalization
0.823	2.02 ± 1.11	2.00 ± 1.01	2.01 ± 1.12	GOS

## Discussion

Various drugs led to neuroprotection in TBI in animals or human models. Only a limited study has explored the influence of amantadine after TBI. The studies about zolpidem are insufficient too (10, 11). In a study in Slovakia, patients took amantadine on day three of admission. Their results proposed that in these objects, the ending GCS was higher and the death rate was inferior to the patients treated with ordinary treatment only (38). After that, in a parallel study, researchers observed the influence of amantadine and placebo in 184 patients who were in a vegetative state and detailed that during the one-month treatment course, recovery was meaningfully quicker in amantadine group (27).

Additional related studies state that after a TBI, amantadine is a realistic choice for improving cognition and decreasing agitation; but, the positive data for the effectiveness of it is required. Both of the above-mentioned studies recognize the probable effect of amantadine on neural utilities in TBI patients, especially DAI and indorse authenticating tools (10, 39, and 40). Similar studies conducted on zolpidem effect on acute traumatic patient are insufficient. Whyte et al. surveyed the rate of substantial clinical response to zolpidem in patients with conscious disorder. Of the 15 patients who were in a vegetative state, at least 1 month after their brain injury, only 1 (7.6%) patient presented a significant clinical response to zolpidem. The clinical response was to diminish the patient's vegetative state (41). Another study by ‌Bo Du et al. in 2013 examined the effect of zolpidem on 165 patients in the post-traumatic vegetative state. According to this study, zolpidem is an effective drug in restoring brain function in patients with a vegetative state after brain injury, particularly those with brain damage in an area other than the brainstem. Improvement in brain function in these patients had more sudden scenario than gradual (42).

A 2014 study by Chatelle et al. surveyed the effect of zolpidem on three patients’ postoperative cognition disorder after chronic anoxia. Unlike the previous study, the effect of zolpidem has been found to be inconsistent. All three patients recovered in terms of communication function, and positron emission tomography (PET) results displayed an increase in the metabolism of the prefrontal dorsolateral and cortical mesioferontal areas after the administration of zolpidem, compared to the control group (34). In another study by Snyman et al. on traumatic children, zolpidem tends to reduce the response in children with stable plant status. It seems that using zolpidem in this situation was not a good option (43).

However, in our present study, patients’ consciousness gradually increased with zolpidem compared to the placebo group, in fact, zolpidem increased the coma scores especially on the fourth day compared to the placebo group but not effective than the amantadine group. Eventually, distinct the above-mentioned studies, there were no important differences between 3 groups based on GOS-GCS, mortality, length of hospitalization in ICU, and duration of mechanical ventilation in our study. But, the results of this study show that patients' GCS increases more and faster in the amantadine groups compared to the 2 other groups, especially after the 4^th^ day of administering it. Results revealed to us that the patients had experienced a more rapid recovery on amantadine use. 

Change percentage GCS score (between admission and on the 11^th^ day) and Effect size (between admission and day 11) were higher in amantadine group compared to the other 2 groups. It showed the greater effect of amantadine in an increasing GCS in admitted patients. So, in this randomized, controlled trial study comprising patients with acute TBI of consciousness, we believed that the administration of amantadine in an acute phase after injury improved the rate of patients’ GCS and GOS score compared with the zolpidem and placebo groups, but without any significant statistical difference.

It is important to note that mechanisms, receptors, pathways such as the reticular activating system, cortex, and communication leading to alertness are not yet fully understood. Amantadine on NMDA and dopamine receptors has a demonstrated effect, and additional imperative mechanisms are likely to be injured following trauma, which will ultimately affect the outcome of the concerned patients. We determine that amantadine is more effective in quickening recovery in patients with acute severe TBI and disorders in consciousness. Exposure to amantadine is related with faster appearance of the rate of recovery in consciousness improvement. Finally, we may emphasize that in the group of patients with severe brain injuries treated with standard therapy plus amantadine, the outcome GCS was higher lower than in the group treated with a standard therapy alone.

Whether treatment with amantadine, as matched with zolpidem and placebo, progresses the long-term outcome and increases speed recovery of function level remains unknown. 

Our study limitations were unfeasibility of the patients’ follow-up after discharge. We record the patients’ GOS precisely before discharge, because the goal of our study was not a long-term follow-up and some patients were not accessible after discharge. Accordingly, our definitions do not report the effects of protracted management on long-term outcomes.

Despite various studies, our results emphasized that amantadine has some valuable effects on the consciousness level of patients with acute TBI compared to other administered drugs like zolpidem. So, upcoming investigation should be given emphasis on defining the pathophysiological features of patients who have a response to amantadine and other neuroprotective drugs, the most effective dosage and duration of treatment and timing of their administering, and the efficacy of amantadine in patients with non-traumatic brain injuries. Consequently, we suggest that further studies with regard acute brain trauma will accomplish other considered medications like methylphenidate.

Traumatic brain injury (TBI) sometimes is a disastrous experience that has disturbing familial and societal penalties. Agreeing to the results of prior studies and this study, it can be detailed that amantadine and other neuroprotective drugs are still used consistently in some centers in patients with DAI and altered consciousness state. 

But since amantadine has intense biochemical effects on many pathways, it looks valuable in an acute period after DAI-associated TBI. Despite various studies, our results emphasized that amantadine has some valuable effects on the consciousness level of patients with acute TBI compared to other administered drugs like zolpidem. We advise that further studies on this topic should be carried out with considered medications like methylphenidate.

## Funding:

This study was a Neurosurgery specialty thesis of Dr. Saeed Aghapour financially supported by Mazandaran University of Medical Sciences, North of Iran and was approved by the Clinical Research Development unit of Imam Khomeini Hospital, MAZUMS.

## Conflict of Interest:

None declared.
